# A Low-Power CMOS Wireless Acoustic Sensing Platform for Remote Surveillance Applications

**DOI:** 10.3390/s20010178

**Published:** 2019-12-28

**Authors:** Yong Wang, Ranran Zhou, Zhenyue Liu, Bingbo Yan

**Affiliations:** School of Microelectronics, Shandong University, 1500 Shunhua Road, Jinan 250101, China; zhenyueliu@mail.sdu.edu.cn (Z.L.); bby@mail.sdu.edu.cn (B.Y.)

**Keywords:** wireless acoustic sensing, remote surveillance, low-power, CMOS

## Abstract

A low-power wireless acoustic sensing platform for remote surveillance applications based on a 180 nm CMOS technology is proposed in this paper. The audio signal, which is acquired by a microphone, is first amplified and filtered. Then, the analog signal is converted to a digital signal by a 10-bit analog-to-digital converter (ADC). A digital automatic gain control module is integrated to obtain an optimal input of the ADC. The digital signal is modulated and transmitted at the 433 MHz ISM band after being repacked and encoded. To save power for portable applications, the chip switches to standby mode when no audio is detected. The wireless sensing platform occupies a chip area of 1.76 mm${}^{2}$. The supply voltage is 2.5 V for the power amplifier and 1.8 V for other circuits. The measured maximum output power is 5.7 dBm and the transmission distance is over 500 m for real application scenarios. The chip consumes 25.1 mW power in normal work mode and 0.058 mW in standby mode. Compared to existing wireless acoustic sensors, the proposed wireless acoustic sensing platform can achieve features such as compactness, power efficiency, and reliability.

## 1. Introduction

Remote sensing systems, which aim to monitor certain kinds of information from a distance, affect the ways people live and work. Wireless sensing platforms such as structural health, bridge, temperature, vision, and human body sensors have been proven to be useful in many monitoring systems [1,2,3,4,5]. Among various kinds of remote sensors, wireless acoustic sensor can be especially useful. They can be employed in application scenarios including indoor acoustic surveillance, crime prevention and investigation, public square and car park monitoring tasks, and so on [6,7,8,9,10].

In the literature, several remote acoustic monitoring systems have been introduced. In one noise monitoring application, the microcontroller unit for signal processing and the wireless transceiver for data acquisition were integrated into one printed circuit board (PCB), where the sampled data were first processed by a microprocessor and then transmitted by a ZigBee module [10]. An ARM-embedded multicast scheme for multiuser speech communication system has been reported in [11], which uses wireless routers to transmit data. Although the above-mentioned systems can collect acoustic information remotely, they are not suitable for portable usage because of their large physical sizes and high power consumption. For easy to carry and easy to install purposes, a wireless remote acoustic sensing platform is often preferred to be light, small, and power efficient. To scale down the device size as well as the power consumption, developing a remote acoustic sensing platform using a single integrated circuit (IC) chip is a better choice.

In the literature, radio frequency transmission practices for wireless acoustic sensing platforms include frequency modulation (FM) [12,13] and carrier-free impulse radio, such as ultra-wideband (UWB) [14,15]. Among them, the UWB approach has the highest power efficiency; however, its transmission range is usually limited to be less than 10 m. Although the analog FM modulation approach [12,13] has been proven to be a feasible solution for mid-range wireless acoustic signal sensing, it is not suitable for applications where the transmitted signals need to be encrypted. To achieve acoustic data encryption for mid-range wireless transmission, digital modulations such as frequency-shift keying (FSK) or phase-shift keying (PSK) are needed. Meanwhile, digitization of the analog acoustic signal also simplifies the acquisition of input signal statistics which is needed for the standby mode control feature. Despite the above-mentioned merits, an IC-based acoustic sensor with digital FSK/PSK modulation has not been reported in the literature.

This paper proposes a monolithic integrated wireless acoustic sensing platform for remote surveillance applications. The chip integrates an analog module for acoustic signal sensing, a digital module for data processing and control, and a RF module for wireless data transmission.

Compared to existing wireless acoustic sensors, the key contributions of this work are listed as follows:

- Designed a binary/differential phase-shift keying (BPSK/DPSK) digital modulation -based wireless acoustic sensing IC, which has better noise immunity than the analog modulation approaches and can achieve data encryption feature.
- Developed an on-chip standby mode control to achieve better battery efficiency. A smart power control strategy makes it possible for the sensor to achieve a long transmission range as well as a long battery life. It is a more integrated approach than the off-chip microcontroller-based standby mode implementation reported in [16].

In summary, an acoustic sensing system based on the above chip can achieve features such as compactness, power efficiency, and reliability. Thus, it is suitable for portable—especially wearable—applications.

The remainder of this paper is organized as follows: Section 2 presents the system design of the proposed wireless sensing platform. Section 3 describes the design of the microphone amplifier and automatic gain control. Section 4 describes the frequency synthesizer with passive temperature compensation techniques. Section 5 gives the PA design. The test circuits and measurement results are given in Section 6. Section 7 concludes the paper.

## 2. Block Diagram

**Figure 1:**
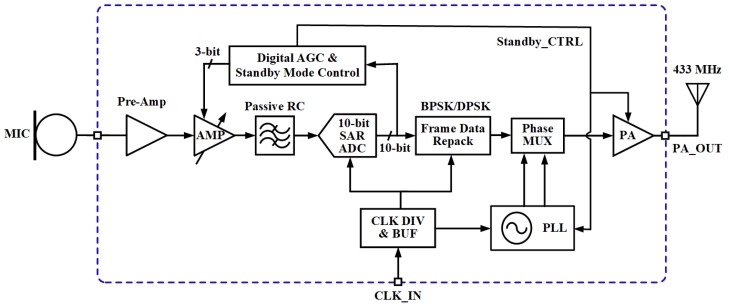
Block diagram of the proposed acoustic sensing platform.

The block diagram of the proposed acoustic sensing platform is illustrated in Figure 1. The analog audio signal captured by the microphone is first amplified by the microphone amplifier, which includes a pre-amplifier (Pre-Amp) and a programmable-gain amplifier (AMP), and then is filtered by a passive low-pass filter (LPF) realized by a RC network. A 10-bit successive approximation type analog-to-digital converter (SAR ADC) is designed to realize analog audio signal to digital signal conversion. The envelope of the digital signal is sampled and fed to a digital automatic gain control (AGC) block to calculate a proper gain for the AMP dynamically to compensate for variations in level from the input of the microphone.

The digital output data is repacked and encoded into frames. The digital data selects a pair of differential local oscillating (LO) signals with opposite phases to realize BPSK/DPSK modulation. The modulated signal is then fed to a class-E power amplifier (PA) and transmitted by an antenna. The LO signals are generated by a phase-locked loop (PLL) with a three-stage ring voltage-controlled oscillator (VCO) to scale down the area of the chip.

The PLL and PA are the energy-hungry parts in the proposed architecture. To achieve high energy efficiency, the system enters standby mode—in which the PLL and PA are turned off—when no audio signal is detected for a certain period of time. In standby mode, the standby control circuits are always on, the microphone amplifier and the ADC will be turned on for 1 ms in every 100 ms. During the 1 ms turn-on time, the microphone amplifier will be adjusted to maximum gain and the control module will check if there is an acoustic signal detected at the microphone. The system will be powered on to full operation mode if an acoustic signal is detected; otherwise, the standby mode will continue.

**Figure 2:**
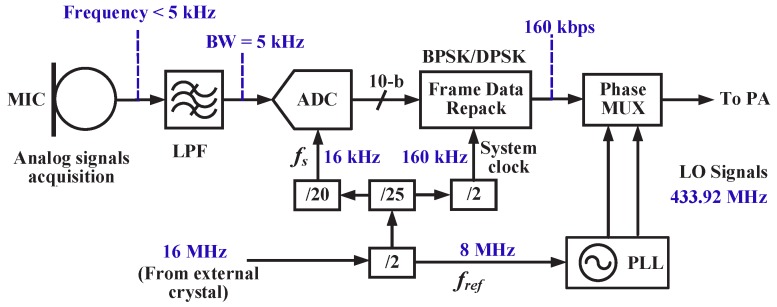
Frequency planning.

The acoustic transmitter operates in the 433 MHz ISM band. The frequency plan is shown in Figure 2. The frequency of the target audio signal captured by the microphone ranges from 300 Hz to 3.4 kHz. As a result, the cut-off frequency of the LPF is set to 5 kHz and the sampling frequency of the ADC is chosen to be 16 kHz to ensure a relatively good sound quality. The frame data repack module takes the ADC outputs and generates a sequence of bits with a data rate of 160 kbps. The PLL oscillates at 433.92 MHz to provide LO signals with an input reference frequency of 8 MHz. All above system and reference clocks come from an external crystal with a frequency of 16 MHz, using frequency division modules.

## 3. Microphone Amplifier and Automatic Gain Control

Amplifiers with high gain are used to amplify the output of a micro-electro-mechanical system (MEMS) microphone. The sensitivity of a MEMS microphone usually ranges from −50 to −38 dBV/Pa [17]. The sensitivity is typically calculated by

(1) $$Sensitivity\;\left[\mathrm{dBV}/\mathrm{Pa}\right]=20\log\frac{Sensitivity\;[\mathrm{mV}/\mathrm{Pa}]}{1000\;\mathrm{mV}/\mathrm{Pa}}.$$

The loudness of a sound is described by the sound pressure level (SPL), which is defined as

(2) $$SPL\;\left[\mathrm{dB}\right]=20\log\frac{P_{m}}{P_{o}},$$

where $P_{m}$ is the measured sound pressure and $P_{o}$ is the threshold of hearing, usually 20 μPa. The sound pressure levels of the various sound sources we are interested in are in the range of 62–42 dB SPL, which corresponds to sound pressure for normal conversion at 1–10 m [18]. Considering the loss of passive RC filtering and the PVT variations to be 3 dB and the microphone sensitivity variation to be 12 dB, a dynamic gain range of 35 dB is sufficient for the microphone amplifier.

With a sensitivity of −50 dBV/Pa and a minimum sound pressure of 42 dB SPL, the voltage level of the acoustic signal at the microphone output is 7.96 μVrms. To amplify this voltage to the desired 600 mVp input range of ADC with a supply voltage of 1.8 V, a total of 98 dB gain is enough, taking the loss and variation of 3 dB into consideration.

**Figure 3:**
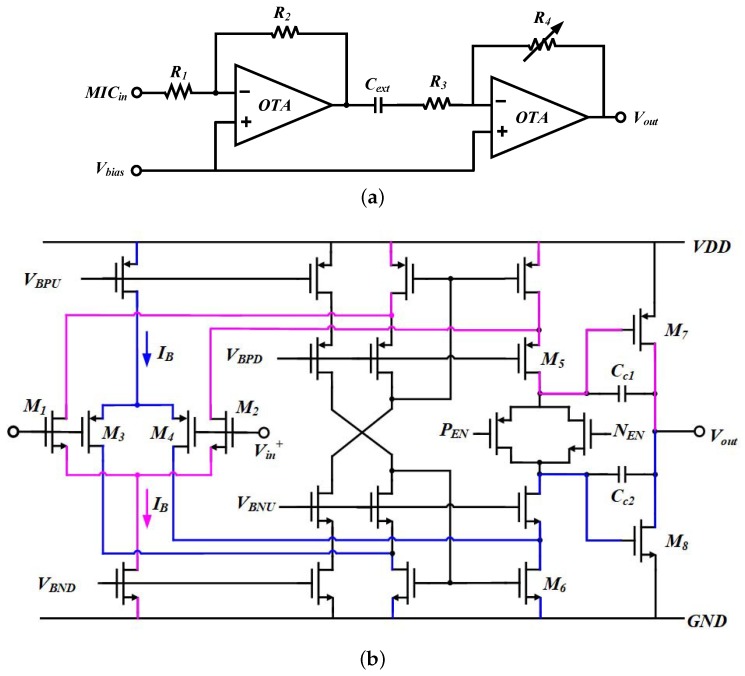
Schematics of (**a**) the two-stage microphone amplifier; and (**b**) each two-stage operational amplifier (OTA).

As Figure 3a shows, the microphone amplifier includes a fixed gain pre-amplifier and a programmable-gain amplifier, coupled through an off-chip capacitor $C_{ext}$. The first stage amplifier is designed with a constant gain of 50 dB, while the gain of the second stage ranges from 6–48 dB with a 6 dB step size, programmed by the AGC loop according to the input level of the audio signal.

Figure 3b gives the detailed schematic of the two-stage operational amplifier (OTA). The input stage with a double-folded cascade topology is designed to achieve rail-to-rail input [19,20]. As a result, the equivalent transconductance is improved by a factor of 2 and the 1/*f* noise can be suppressed notably [20]. The output stage is comprised of two complementary transistors and two Miller capacitors to realize rail-to-rail output. The gain-bandwidth product (GBP) of the OTA is set to 6.8 MHz.

The AGC loop adjusts the level of the input applied to the ADC when the level of the audio source is unknown. The target level for the output is set such that any transients on the microphone input will not clip during normal operation. The AGC circuit compares the output of the ADC to this target level and increases or decreases the gain of the AMP to compensate.

**Figure 4:**
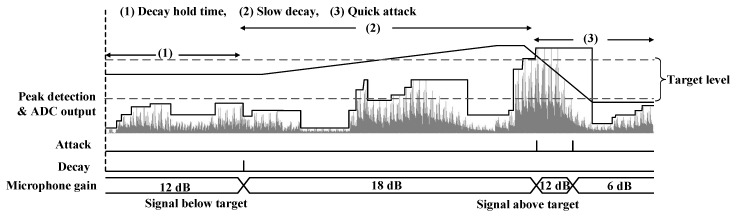
Principle of microphone automatic gain control (AGC) loop.

The principle of the AGC loop is illustrated in Figure 4 [21]. The ADC output signal is initially below the low threshold of the target level. After the hold time has finished, the gain of the AMP is increased at a programmable rate ((1) → (2)). Later, the amplitude of output signal increases and reaches the high threshold of the target level. The AGC is programmed to decrease the gain at a faster rate ((2) → (3)), to allow the elimination of typical popping noises.

Since the amplitudes of acoustic signals carry information, AGC control circuits are designed to respond only to the statistical sound level of a frame of the input audio signal. As illustrated in Figure 4, the relative amplitudes of the original sound are kept unchanged inside a frame of sound data. The gain is adjusted only at the boundary between two frames. There is a delay between when the AGC attenuates the ADC input and when it reads the ADC output. During this delay, the ADC input signal may clip if there is not enough headroom available. The headroom should be approximately the difference between the average signal level and the level of the expected transients [21]. On the other hand, the digital AGC algorithm relies on a user-specified extra latency to avoid over amplifying the background noises during the pause period of an audio signal.

## 4. Frequency Synthesizer with Temperature Compensation

**Figure 5:**
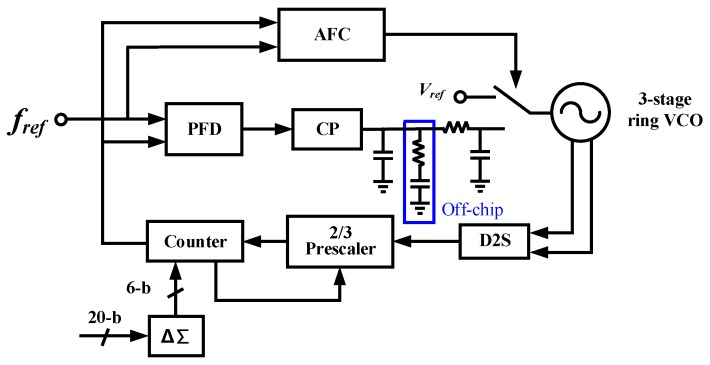
Phase-locked loop (PLL) block diagram.

Figure 5 shows the block diagram of the frequency synthesizer, which is realized by a fractional-N PLL [22]. The PLL consists of a phase frequency detector (PFD), a charge pump (CP), a LPF, a 3-stage ring VCO, a differential-to-single-ended (D2S) module, a divider, and an automatic frequency calibration (AFC) module. The divider includes a 2/3 prescaler, a programmable counter, and a delta-sigma (ΔΣ) module.

The PLL powers on when the control module detects an acoustic signal. First, the frequency tuning words are initiated, the PLL loop is set to open and the VCO input voltage connects to a voltage reference. Then the AFC compares the VCO output frequency with the reference clock and sets the proper divider value, as well as the VCO coarse frequency control words (FCW). Once an automatic calibration process is done, the PLL loop will be closed and a normal locking process will start.

**Figure 6:**
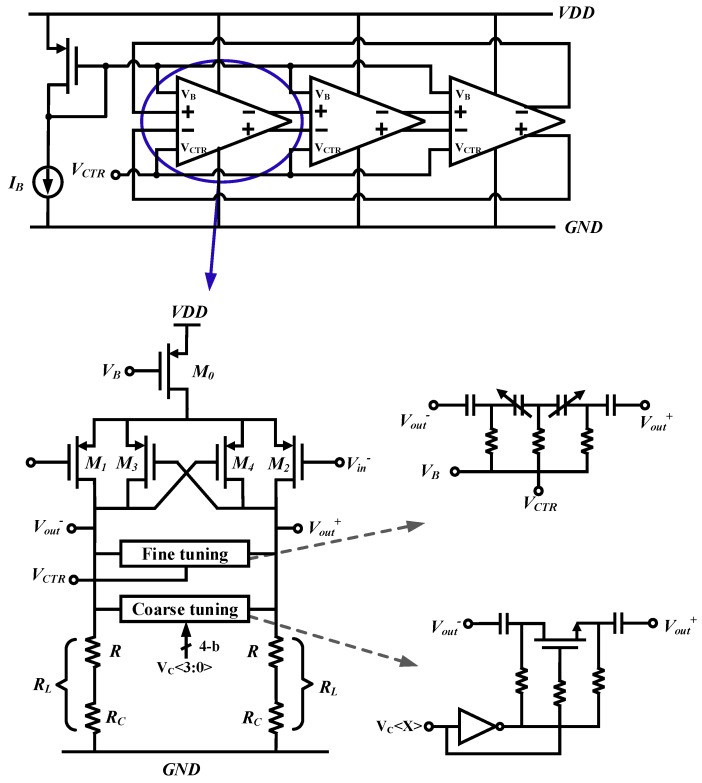
Schematic of the ring voltage-controlled oscillator (VCO).

The schematic of the 3-stage ring VCO is shown in Figure 6. To achieve a wide tuning range for the VCO with a low sensitivity to noise, a VCO cell typically consists of coarse tuning circuits implemented with a switched capacitor bank and fine-tuning circuits done with varactors. A 4-bit control word is used to carry out coarse calibration against PVT variations during the circuit initialization stage. The fine-tuning will take effect when the PLL enters the normal locking process. The target oscillating frequency *f* of the VCO can be calculated by

(3) $$f=\frac{1}{2\pi }\left(\tan\frac{180^{\circ }}{N}\right)\frac{1-g_{m2}R_{L}}{R_{L}C_{L}},$$

where *N* is the number of VCO stages, $g_{m2}$ is the transconductance of $M_{2}$, and $R_{L}$ and $C_{L}$ are the resistance and capacitance of the load resistor and capacitor, respectively.

In the chosen technology, the resistance of the normal resistors (often P-poly type) is proportional to the temperature with a negative temperature coefficient (TC). Once the PLL is started, frequency drifts caused by extreme temperature variations could push the control voltage of the VCO out of the fine-tuning range, causing the PLL to become out of lock. Although temperature-related frequency drifts can be compensated by schemes such as tuning the tail current [23,24] and adding extra compensation loops [25], these methods lead to complex designs and extra area. To compensate the temperature drift with minimum circuitry, an N-well resistor which exhibits positive TC is employed. The load resistor $R_{L}$ can be implemented as a series combination of a P-poly resistor *R* and an N-well compensation resistor $R_{C}$. The combined TC of the complex resistor can be derived by

(4) $$TC_{L}=\frac{R_{C}}{R_{L}}TC_{C}+\frac{R}{R_{L}}TC,$$

where $R_{L}=R_{C}+R$ and $TC_{L}$, $TC_{C}$, and TC are temperature coefficients of $R_{L}$, $R_{C}$, and *R*, respectively.

Suppose $g_{m2}$ changes by $\Delta g_{m2}$ when temperature changes by $\Delta T=T-T_{0}$. With Equation (3), the frequency drift can be written as

(5) $$\begin{array}{cc} \Delta f & =\frac{1}{2\pi }\left(\tan\frac{180^{\circ }}{N}\right)\left[\frac{1-\left(g_{m2}+\Delta g_{m2}\right)R_{L}\left(1+TC\cdot \Delta T\right)}{R_{L}\left(1+TC\cdot \Delta T\right)C_{L}}-\frac{1-g_{m2}R_{L}}{R_{L}C_{L}}\right]\\ \; & =\frac{1}{2\pi }\left(\tan\frac{180^{\circ }}{N}\right)\left[\frac{1}{R_{L}\left(1+TC\cdot \Delta T\right)C_{L}}-\frac{1+\Delta g_{m2}R_{L}}{R_{L}C_{L}}\right]. \end{array}$$

If a linear approximation $\Delta g_{m2}=\alpha \Delta Tg_{m2}$ is adopted, let the drift frequency Δf be equal to 0. Then, we can get

(6) $$\frac{1}{1+TC\cdot \Delta T}=1+\alpha g_{m2}\cdot R_{L}.$$

Combining Equations (4) and (6), to derive a minimum drifting frequency Δf, the resistances of $R_{C}$ and *R* can be calculated as

(7) $$R_{C}=R_{L}\frac{\frac{1}{\Delta T+\alpha g_{m2}\Delta T^{2}\cdot R_{L}}-\frac{1}{\Delta T}-TC}{TC_{C}-TC},$$

(8) $$R=R_{L}\frac{\frac{1}{\Delta T+\alpha g_{m2}\Delta T^{2}\cdot R_{L}}-\frac{1}{\Delta T}-TC_{C}}{TC-TC_{C}}.$$

**Figure 7:**
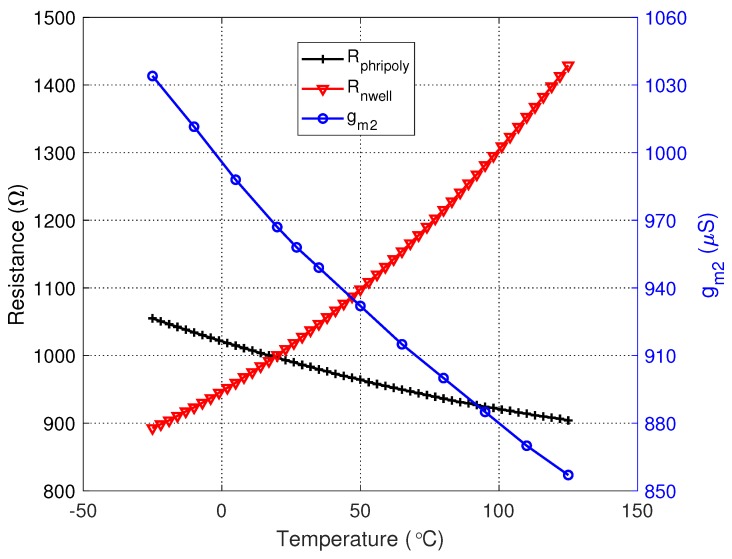
Resistances of $R_{phripoly}$, $R_{nwell}$, and transconductance of $M_{2}$ versus temperature.

As shown in Figure 7, the value of $g_{m2}$ decreases from 1034 μS to 853 μS over −25 to 125 ${}^{\circ }$C, and the temperature coefficients of a phripoly-type resistor and an N-well-type resistor are of opposite signs. By choosing proper values of *R* and $R_{C}$, the frequency drift caused by temperature variation can be minimized.

## 5. Class-E PA

**Figure 8:**
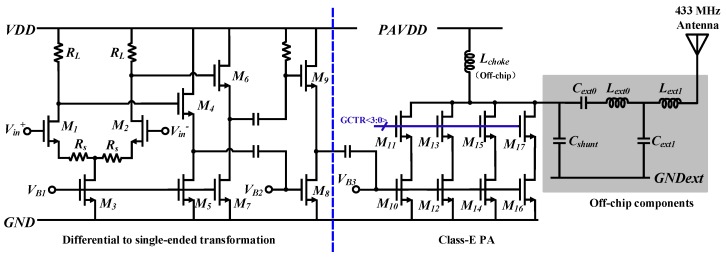
Schematic of class-E PA.

To achieve high power efficiency, a class-E PA with cascade topology [26,27,28] is designed as the output stage. As Figure 8 shows, the PA consists of a buffer which is employed to realize differential to single-ended transformation, four switched cascade branches to enhance the robustness and adjust the output power, an off-chip choke inductor $(L_{choke}$), and an off-chip shunt capacitor ($C_{shunt}$) to determine the operating frequency, as well as an off-chip matching network including $C_{ext0}$, $L_{ext0}$, and $L_{ext1}$.

To verify the performance of the PA, a post-layout simulation was carried out. In this simulation, the supply voltage of the PA was set to be 2.5 V, which is higher than the other circuits, whose supply voltage was 1.8 V. The PA had a simulated maximum output power of 7 dBm and demonstrated a power-added efficiency (PAE) of 62%.

## 6. Test Circuits and Measurement Results

**Figure 9:**
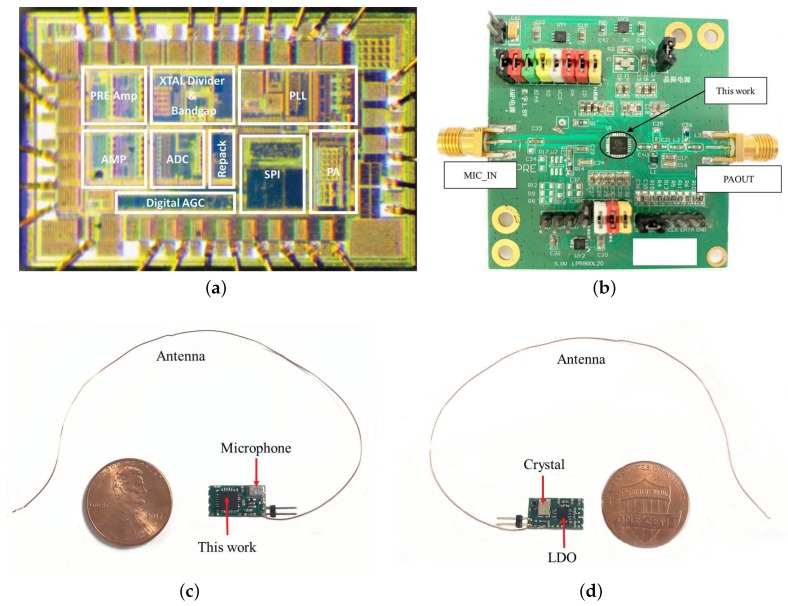
(**a**) Chip microphotograph; (**b**) testing board; (**c**) a demo compared with a coin (front); and (**d**) a demo compared with a coin (back).

The proposed wireless acoustic sensing platform was implemented in a 180 nm CMOS technology. The chip microphotograph is shown in Figure 9a. The whole die area is approximately 1.68 mm × 1.05 mm. The measurements were carried out after chip packaging and the testing PCB is designed as Figure 9b. To further verify the performance of the acoustic sensing platform, a compact application demo was designed, as is shown in Figure 9c,d, which was implemented in a four-layer PCB with a size of 1.26 cm × 0.72 cm weighing less than 1 g without the battery. The demo based on the proposed platform included the packaged chip, a microphone, a crystal, an LDO, an antenna, and peripheral components to connect to a 2.5 V battery. The antenna was realized by a copper wire whose length was 1/4 wavelength (λ) of the 433 MHz electromagnetic wave. Here, $\lambda =c/f=3.0\times 10^{8}/\left(433\times 10^{6}\right)$ m = 69.3 cm. As a result, the length of the copper wire antenna was designed as 17.3 cm.

**Figure 10:**
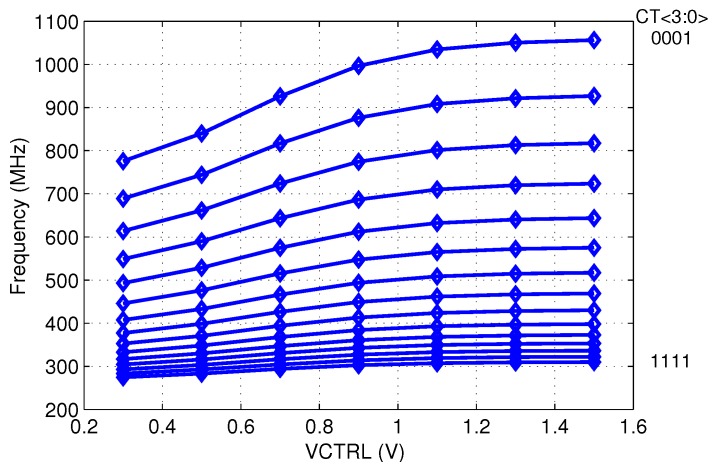
The measured output frequency of the VCO.

**Figure 11:**
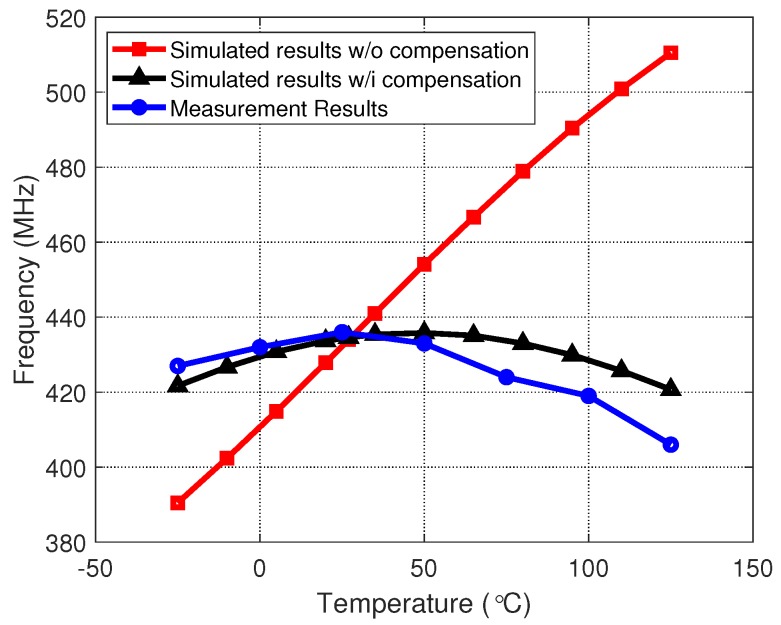
Frequency variations versus temperature with/without compensation.

Figure 10 shows the measured output frequency curves of the VCO. The oscillating frequency of the VCO ranged from 290 MHz to 1.04 GHz. The simulated and measured output frequency variation of the VCO over −25 to 125 ${}^{\circ }$C temperature range are shown in Figure 11. Without compensation, a simulated temperature sensitivity of 1874 ppm/${}^{\circ }$C along with a 121 MHz frequency variation can be observed over the full temperature range. With the above-mentioned compensation scheme, temperature-related output frequency variations over the full temperature operating range were reduced to around 15 MHz in post-layout simulation and about 20 MHz in measurement. The average temperature sensitivity was reduced to 307 ppm/${}^{\circ }$C, which is 16.38% of the original circuit. Although a design using the above method had a relatively higher frequency coefficient, compared to the current compensation approaches [23], it is area-efficient and suitable for low-cost applications. A performance summary is given in Table 1.

**Figure 12:**
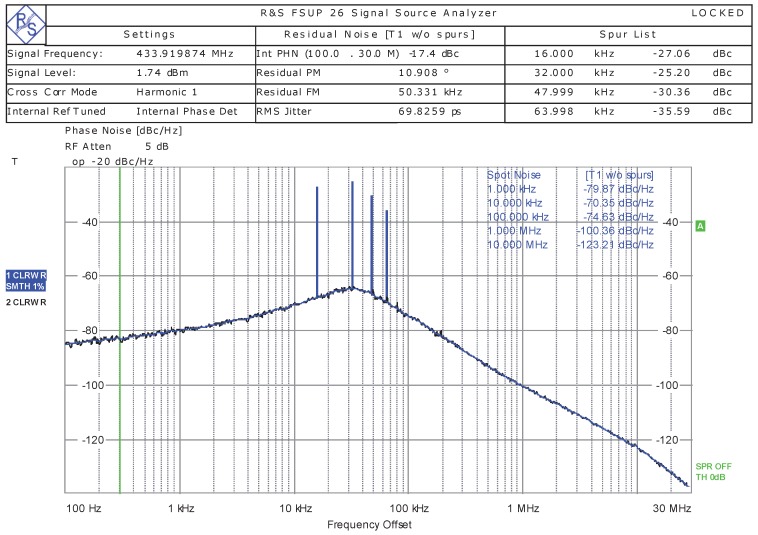
The measured phase noise of the PLL.

Figure 12 shows the measured phase noise of the PLL. From the measurement results, it can be seen that the bandwidth of the PLL was about 100 kHz and the phase noise was −100.36 dBc/Hz at a 1 MHz frequency offset.

**Figure 13:**
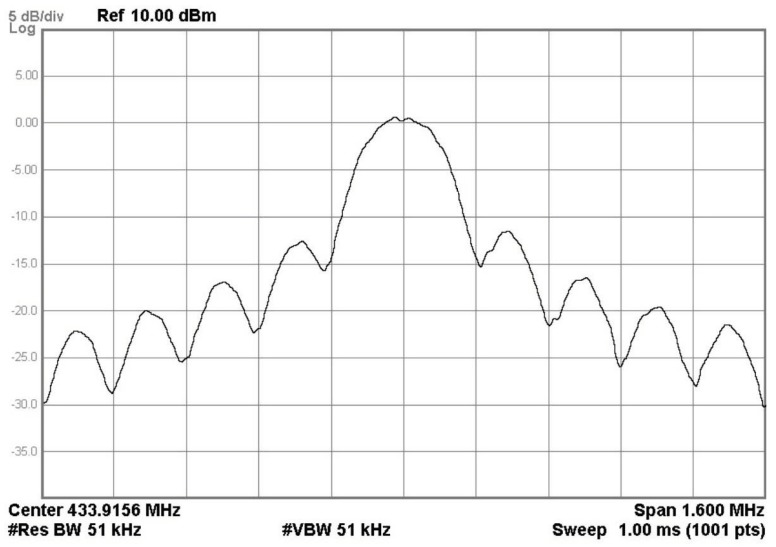
The measured 160 kbps binary phase-shift keying (BPSK) spectrum.

With a 1 kHz, 1 mVpp AM sinusoidal signal at the microphone input port, the output BPSK spectrum with maximum output power is shown in Figure 13. The operating frequency was 433.916 MHz. The signal bandwidth was 320 kHz and the side-band components were suppressed by more than −13 dBc. A channel power of 5.7 dBm can be measured by the spectrum analyzer.

**Figure 14:**
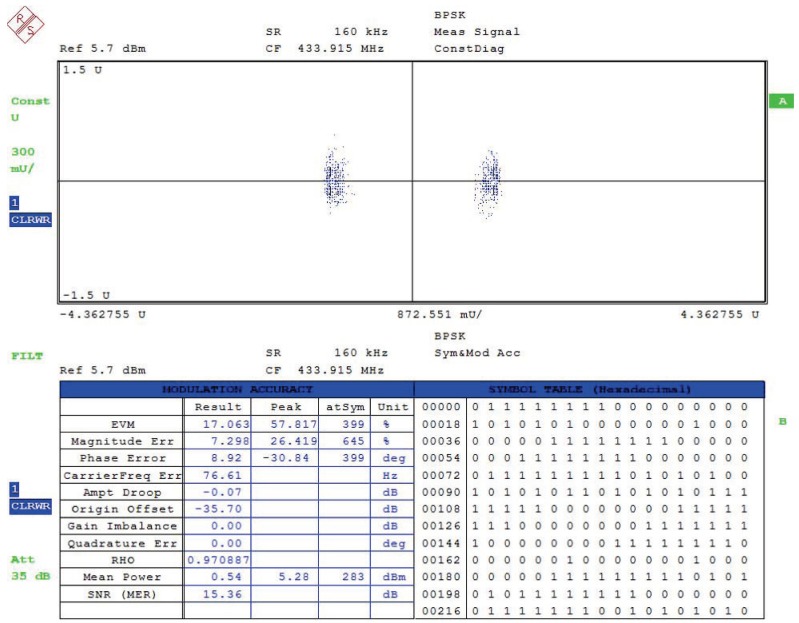
Measured error vector magnitude (EVM).

The measured error vector magnitude (EVM) is shown in Figure 14. With 160 kbps BPSK data modulated at 433.92 MHz, the measured EVM was about 17.06% rms. The magnitude error was 7.298% and the phase error was 8.92${}^{\circ }$. The figure also shows the measured SNR, which was about 15.36 dB.

**Figure 15:**
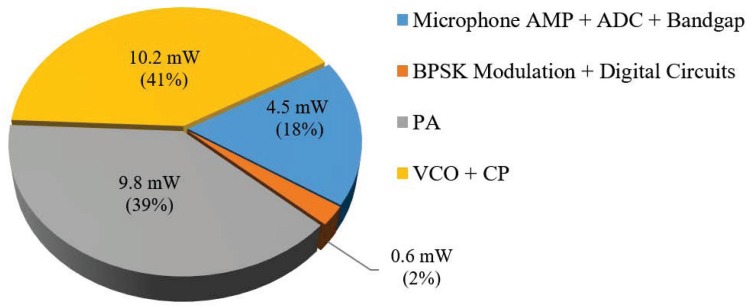
Measured power consumption.

Each major block of the chip can be powered on/off individually using one control bit via the on-chip serial peripheral interface (SPI). As a result, the power consumption of each block can be measured. The power consumption distribution for various circuit blocks is shown in Figure 15. The total power consumption for the chip was about 25.1 mW with the output PA working at its maximum gain. The microphone amplifier and analog parts of the ADC, as well as bandgap, consumed 4.5 mW in total. The BPSK modulation circuits and the digital circuits of AGC, ΔΣ, and so on, had a power consumption of 0.6 mW. The energy-hungry VCO and PA consumed about 20 mW, approximately 80% of the total power consumption. Off-chip components including a reference oscillator, an LDO and a microphone consume 6.55 mW, thus the power consumption for the demo board shown in Figure 9c,d was 31.65 mW. The on-chip system power consumption for standby mode is 0.058 mW, which is dramatically smaller than the power consumption of the normal mode.

**Figure 16:**
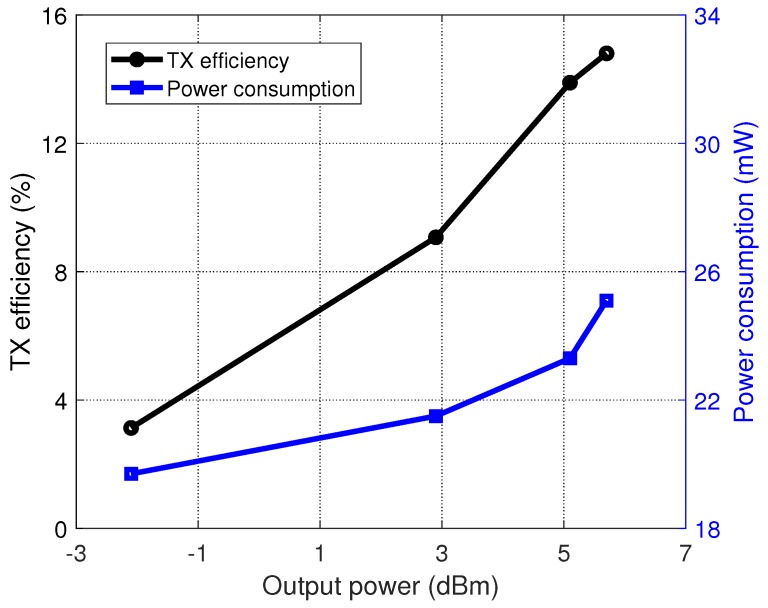
Measured whole TX efficiency.

Figure 16 shows the efficiency characteristic of the whole transmitter. When operating at 433.92 MHz, the transmitter efficiency was about 14.8% with the maximum output power.

To verify the system performance of the demo board, a receiver with −109 dBm sensitivity was designed using commercial chips. Assuming the antenna attenuation to be 6 dB and the obstacles along the transmission path, such as walls, trees, and so on, to contribute another 25 dB loss, the free space attenuation limitation is 83.7 dB for a sensing platform with a 5.7 dBm maximum output power. According to the propagation loss of radio waves in free space [29], the propagation loss ($L_{fs}$) can be written as

(9) $$L_{fs}\left[\mathrm{dB}\right]=32.44+20\log d\left[\mathrm{km}\right]+20\log f\left[\mathrm{MHz}\right],$$

where *d* is the transmission distance and *f* is the operating frequency, respectively. With a free space propagation loss of 83.7 dB and a working frequency of 433.92 MHz, the transmission distance is calculated to be 842 m. In our experiment, we verified that the effective transmission distance was over 500 m with a concrete wall, a glass window, and a row of high trees between the transmitter and the receiver.

The performance of the proposed acoustic sensing chip is summarized in Table 2.

## 7. Conclusions

A highly integrated wireless acoustic sensing platform was presented in this paper. The platform has transmission data encryption and standby mode control features and is for remote surveillance applications. The chip was fabricated in a 180 nm CMOS technology and the tested maximum output power was 5.7 dBm with an effective transmission distance of over 500 m. The chip consumed 25.1 mW in normal working mode and 0.058 mW in standby mode. The compactness and power efficiency of the acoustic sensing platform make it suitable for various remote surveillance application scenarios.

## Figures and Tables

**Table 1:** Performance summary of the VCO temperature compensation.

Parameter	[23]	[24]	[25]	This Work
Type	Ring	Ring	Ring	Ring
Technology [nm]	180	130	90	180
Supply voltage [V]	3	1.1	1	1.8
Frequency [MHz]	1.9	1250	2150/2900	433.92
Area [mm${}^{2}]$	0.22	0.014	0.096	0.044
Temperature range [${}^{\circ }$C]	−40–80	−40–125	−7–67	−25–125
Temperature sensitivity [ppm/${}^{\circ }$C]	92.8	340	168/290	307

**Table 2:** Performance summary and comparison.

Parameter	[12]	[14]	[16]	This Work
Technology	0.25 μm BiCMOS	1 μm SOI	130 nm CMOS	180 nm CMOS
Physical signal type	Acoustic	Acoustic (high	Temperature/Tilt/	Acoustic
for sensing		temperature)	Acceleration	
Operating frequency [MHz]	900	1.4	1900	433.92
Modulation	FM	OOK/Chirp	OOK	BPSK/DPSK
Standby Mode	No	No	Yes (off-chip)	Yes (on-chip)
Supply voltage * [V]	1.8–2.8	5	0.65	1.8 (2.5 for PA)
VCO tuning range [MHz]	891–939	1.376–1.424	N.A.	290–1040
PLL phase noise	N.A.	N.A.	N.A.	−100.36 dBc/Hz
				@ 1 MHz offset
Output power [dBm]	3	N.A.	0.8	5.7
Area * [mm${}^{2}]$	1.215	27.5	1	1.76
Power consumption * [mW]	26	12–19	1.35	Normal mode: 25.1
				Standby mode: 0.058
Transmission distance	N.A.	N.A.	N.A.	>500 m

* Only on-chip parts.
